# Supplementary material for: Are carbon emissions trading and green financial instruments synergistic? -Comprehensive quantitative research based on content analysis
Source: PLoS One. 2024 Mar 7;19(3):e0298601. doi: 10.1371/journal.pone.0298601 (PMC10919601; doi:10.1371/journal.pone.0298601)
Supplement: S1 Data — (ZIP) [file pone.0298601.s001.zip › Data/地级市Z_F工作报告.zip!/┴╔─■/╠·┴δ╩╨/└┤╫╘╣1⁄2╓┌║┼í╛┬φ┐╦╩2╛▌═°í┐_2019.docx]

各位代表：

现在，我代表市人民政府向大会报告工作，请予审议，并请市政协委员和列席同志提出意见。

一、2018年工作回顾

2018年是贯彻落实党的十九大精神开局之年，是改革开放40周年，也是铁岭经济社会发展极不寻常的一年。我们在省委、省政府和市委的坚强领导下，坚持以习近平新时代中国特色社会主义思想为指导，全面贯彻党的十九大和十九届二中、三中全会精神，深入落实习近平总书记在辽宁考察时和在深入推进东北振兴座谈会上的重要讲话精神，坚持稳中求进工作总基调，按照高质量发展要求，持之以恒落实新发展理念和“四个着力”“三个推进”，以供给侧结构性改革为主线，坚决打好“三大攻坚战”，扎实开展“重实干、强执行、抓落实”专项行动，统筹推进改革发展稳定各项工作，全市经济呈现筑底企稳、稳中向好的发展态势，社会保持和谐稳定。

预计，全市地区生产总值完成600.8亿元，增长1%；固定资产投资116亿元，增长11.1%；一般公共预算收入52.2亿元，增长6.2%；规模以上工业增加值115.3亿元，增长5.1%；社会消费品零售总额440亿元，增长0.5%；城乡居民人均可支配收入分别达到24854元和14247元，均增长6.5%。

—— 经济运行质量有所提升

现代农业发展势头良好。积极克服严重旱灾、非洲猪瘟疫情等不利因素影响，加强气象灾害预报预警和人工影响天气工作，全市粮食总产量实现78.4亿斤，肉类总产量56.4万吨。调减籽粒玉米种植面积7万亩，新增高效节水灌溉面积1.5万亩。全市主要农作物综合机械化水平达到88%。新增规模以上农产品加工企业7家。土地规模经营面积达到354万亩。土地托管服务面积30万亩，居全省首位。新发展省级以上农民合作社示范社16个、家庭农场113个。农业标准化生产面积580万亩，“三品”认证达到190个。“铁岭大米”获批国家地理标志保护产品。金正大复合肥等11家企业产品获评省名牌产品。我市获批筹建全国榛子产业知名品牌创建示范区。

工业转型升级步伐加快。新能源产业发展迅速，全市22座风电场、11个光伏发电站和1座生物质发电厂并网发电，总装机容量达129万千瓦。特色装备制造产业加快发展，民生智能仪表等3户企业被评为省级工业设计中心。新增高新技术企业22户，新建省级产业技术创新平台2家。军民融合产业发展取得新进展，中航发动机露天试验平台等5个重点项目签约，铁岭陆平与东软集团合作研制生产的智能移动CT诊疗车正式下线。实施重点技术改造项目36个。新增规模以上工业企业37户，新增产值超亿元企业16户。盘活闲置资产33处、土地996亩、厂房46.5万平方米。

现代服务业平稳发展。铁岭保税物流中心封关运营，首单货物顺利通关。辽宁毕托巴在“新三板”成功挂牌，鑫众科技进入主板上市IPO程序，兴东科技纳入省重点上市企业后备库。中航安盟保险、富邦财险落户铁岭。农村普惠金融示范区建设取得阶段性成果，玉米和大豆“期货+保险”“政银担”合作进展顺利。全市金融机构存贷款余额分别达到1393亿元和906.6亿元。建成农产品电商平台9个，新建省级电子商务示范企业2家。实施42°铁岭品牌培育工程，评选铁岭特色名优品牌产品30个。成功举办各类宣传推介铁岭主题活动30余个。全市旅游业总收入实现166亿元，增长12.1%。

—— 项目年活动扎实推进

“双招双引”一号工程深入实施。开展“走出去、请进来”等招商活动1069次，中慈产业园等185个项目签约，实际到位内资100亿元，增长20.2%。设立了5000万元人才发展专项资金，新增专业技术人才1544人，引进高层次人才16人。

项目建设扎实推进。投资5000万元以上开复工项目118个。调兵山30万吨燃料乙醇项目全线试车投产，昌图30万吨燃料乙醇项目签约，富力地产等一批重大项目进展顺利。包装储备项目140个，列入国家重大建设项目PPP库6个，计划总投资113亿元。向上争取各类政策资金138亿元。

对外开放不断扩大。与淮安对口合作扎实推进，铁岭淮安工业园区项目签约落地。与俄罗斯科学院西伯利亚分院开展合作，搭建了独联体国家科技成果转化落地平台。西丰县鹿产品出口基地、辽宁专用车生产基地分别晋升国家和省级外贸转型升级基地。全市外贸进出口总额分别达到23.6亿元和13.2亿元，增长210.6%和11.8%。实际利用外资3650万美元，增长218.2%。

—— 营商环境建设实现新突破

“最多跑一次”改革成效显著。442项市本级政务服务事项全部实现“最多跑一次”，123项实现当日办结，110个要件实现容缺受理，38个事项办理流程得到优化，平均压缩审批时限42%，审批服务大厅提供无双休日服务，审批效率进一步提升。

“放管服”改革深入实施。企业开办设立、不动产登记、投资建设项目领域“一窗受理”扎实推进。市、县政务服务在线受理事项达到70%。工商登记实现“32证合一”。一般性企业开办时间不超过2.5天，为全省最短。“证照分离”改革全面推开，新登记各类市场主体2.47万户，增长5.8%。

“办事难”问题得到有效解决。先后向社会公布2批、114项解决企业和群众“办事难”新举措。解决群众反映强烈的“办证难”问题实现新突破，全年为124个小区、7万户居民办理了房产证。制定了服务企业十条措施，为204个重点项目配备了“项目管家”，开通了“兴企为民服务直通车”。减税降费政策有效落实，减轻企业电费负担1500万元。

—— 重点领域改革持续深化

国资国企改革取得突破性进展。按照“四个一批”原则，公投、国资等9家市属国有企业集团成功组建。市粮食集团与省粮食集团合资合作取得新进展。央企、省企“三供一业”分离移交完成节点任务。厂办大集体改革稳步实施。

机关事业单位改革顺利实施。政府机构改革如期完成。430家市本级公益性事业单位优化整合为33家。22家市直经营性事业单位完成转企改制。农信社改制等其他领域改革有序推进。

县乡财政管理体制改革全面完成。89个乡镇金库规范运行，全市乡镇级一般公共预算收入完成7.7亿元，增长11.8%。在经济开发区积极复制推广自贸试验区试点经验。工业园区全面实行“管委会+公司”运营模式，管理体制机制进一步理顺。

—— 城乡协调发展统筹推进

乡村振兴战略稳步实施。农村集体产权制度改革有序推进，调兵山市国家级试点工作全面完成。农村土地确权登记工作基本完成，村集体经济不断壮大，新增经营收入3万元以上村239个。先进文化进万家活动深入开展，结成文明单位和文明村共建对子255个。村级文化广场建设实现全覆盖。建成乡镇公共法律服务站30个、村级工作室477个。村规民约修改工作全面完成，基层治理体系进一步完善。培育首批市级特色乡镇16个。

县域经济稳步发展。铁南经济开发区晋升省级经济开发区。调兵山市晓明镇独立工矿区搬迁改造纳入新一轮国家支持范围。“飞地经济”稳步推进，签约落地项目62个，计划总投资101亿元。“大棚房”问题专项清理整治全面完成。土地后备资源开发和提标改造工程稳步推进。县域城区基础设施不断完善，开原滨水新城、昌图滨湖新城建设进展顺利，综合承载能力不断增强。

城乡面貌持续改善。实施棚户区改造970户，货币化安置率100%。备受关注的沙子沟棚户区改造项目全面完工，2200余户居民顺利回迁。单户城镇化、暖房子改造等历史遗留问题有效解决。中心城区主要街路维修改造全面完成，兴工雨污泵站等工程如期竣工。新建改建城市供热、供气等管网65.8公里。完成各级公路建设760公里、一事一议村内道路635.9公里。完成农网升级改造10千伏线路463千米。成功与辽宁铁塔公司签署5G战略合作协议。改造农村危房4581户，新增无害化厕所21650座。培育创建省级生态村镇8个，建设市级美丽乡村示范村47个。

—— “三大攻坚战”扎实开展

重大风险防控工作稳步实施。化解置换地方政府债务73.5亿元。出台断保人员养老保险贴息贷款等支持政策，切实加大养老保险金征管力度，确保了养老金按时足额发放。

精准脱贫工作扎实开展。“五个一批”政策有效落实。实施产业扶贫项目237个。完成贫困户D级危房改造2342户。全面落实建档立卡贫困学生“两免一补”教育扶贫政策，1317名贫困学生就学得到保障。成功开发“收入保”“健康保”扶贫项目，确保了建档立卡贫困人口定点医疗机构住院治疗零负担，旱灾之年稳定脱贫。全年17248名建档立卡贫困人口脱贫、65个贫困村退出和省级贫困县西丰县摘帽任务顺利完成。

污染防治工作强力推进。中央环保督察组反馈任务整改完成9件，信访举报案件完成整改89%。中央环保督察“回头看”下沉发现问题完成整改85%。铁岭县国家畜禽粪污资源化利用整县推进项目启动实施。亮子河、马仲河污染治理初见成效，我市顺利通过国家水生态文明城市建设试点技术评估。拆改10吨及以下燃煤锅炉401台，全年空气质量优良天数达到298天，西丰县获评国家级秸秆综合利用试点县。全市造林面积达22.8万亩。全国第二次污染源普查清查建库工作通过国家评估。

—— 民生和社会事业加快发展

年初确定的15件民生实事基本落实。新增城镇就业1.5万人。城乡低保标准分别提高5.9%和7.4%。城镇居民医保年人均政府补助标准由450元提高到490元，参保患者实现异地就医联网直接结算和即时结报。完成残疾人家庭无障碍改造1150户。市综合性老年人养护中心主体工程完工。双拥优抚安置工作成效显著。

社会事业健康发展。建设连锁幼儿园60所，46所贫困地区义务教育薄弱学校基本办学条件得到改善。全域实现县域内义务教育基本均衡。铁岭师专现代服务业专业群项目落实落地。铁岭技师学院获批为第45届世界技能大赛中国集训基地和国家级高技能人才培训基地。城市医联体和县域医共体加快建设，新建医养结合机构3家，名科、名医、名护创建评选活动有序推进。《大屯小事》等一批优秀剧目在央视舞台展播。

社会治理创新水平不断提高。扫黑除恶专项斗争取得阶段性成果。风险管控与隐患排查治理双重预防机制逐步完善，安全生产形势总体稳定。食品安全隐患排查机制基本建立，市级食品检验检测实验室顺利完工。信访矛盾减存控增三年攻坚计划有效实施，中央巡视组移交的184件行政类信访案件全部办结，人民群众的获得感、幸福感、安全感不断提升。

—— 政府自身建设得到加强

扎实推进“两学一做”学习教育常态化制度化，“解放思想推动高质量发展大讨论”扎实开展，“提士气、鼓干劲、解难题、促发展”主题实践活动成效明显。

“重实干、强执行、抓落实”专项行动全面实施，省市确定的8个方面、206项重点任务有效落实，真正啃下了一批硬骨头，办成了一批难办的事，解决了一批历史遗留问题。

坚持每季度向市委报告工作，主动接受市人大及其常委会法律监督、工作监督和市政协民主监督。认真听取各民主党派、工商联和无党派人士意见。全年办理人大议案、人大代表建议和政协提案527件，满意率达到99%以上，落实率70%以上。

全面从严治党主体责任进一步压实。严格贯彻落实中央八项规定精神，形式主义和官僚主义新表现得到有效遏制。行政权力监督制约机制进一步健全，审计监督、督查考评、应急管理、政务公开工作不断加强。

民族、宗教、外事、侨务、人防、地震、国家安全、烟草、海关、消防、国防动员、仲裁等各项工作也都取得了新成绩。

事非经过不知难。过去的一年，我们在困难中前行，在逆境中拼搏，取得了来之不易的成绩，需要倍加珍惜。这是省委、省政府和市委正确领导的结果，是市人大及其常委会法律监督、工作监督和市政协民主监督与大力支持的结果，更是全市广大干部群众齐心协力、共同奋斗的结果。在此，我代表市人民政府，向辛勤工作在各条战线上的全市各族人民，向积极支持政府工作的市人大代表、市政协委员、各民主党派、工商联、无党派人士、各人民团体、中省直驻铁单位和各界人士，向解放军驻铁部队指战员和武警官兵，向曾经为铁岭振兴发展呕心沥血、奠定扎实基础的老领导、老同志，向所有关心、支持、参与铁岭振兴发展的海内外朋友，表示衷心的感谢并致以崇高的敬意！

在总结工作的同时，我们也要清醒地认识到，面对内外部环境变化，对标高质量发展要求，全市经济社会发展还面临一些突出问题。一是经济总量还比较小。经济增长内生动力还不够足，发展质量和效益不够高，有效投资不足和项目储备不足仍然是经济发展中的突出短板。二是体制机制矛盾突出。改革还不够深入，国企活力不足、历史遗留问题、僵尸企业和民营经济发展滞后问题仍未得到有效解决，法治铁岭、诚信铁岭建设任重道远。三是产业结构调整任务艰巨。特别是产业结构单一，发展新动能没有系统形成，实体经济发展困难较多，人才流失问题比较严重，转方式、调结构任务繁重紧迫。四是开放合作和创新短板突出。对外开放进展不快，主动融入“一带一路”的大格局尚未形成，创新能力不强，特别是科技发展水平还不高，对经济社会发展的支撑能力不足，对经济增长的贡献率还比较低。五是政府职能转变还不到位。有些政策落实不力，一些干部干事创业激情不足，服务意识、法治意识不强，工作作风不实，担当精神不够，存在“多干多错、少干少错、不干无错”的心态，群众还有不少不满意的地方。这些问题需要我们在今后工作中认真加以解决。

二、2019年重点工作安排

今年是新中国成立70周年，是决胜全面建成小康社会的关键之年，也是推进铁岭全面振兴、全方位振兴的攻坚之年，做好各项工作意义重大。我们一定要深刻领会习近平总书记在辽宁考察时和在深入推进东北振兴座谈会上的重要讲话精神，进一步坚定信心，抓住机遇，攻坚克难，开拓创新，奋力谱写铁岭全面振兴、全方位振兴的新篇章。

今年政府工作的总体要求是：坚持以习近平新时代中国特色社会主义思想为指导，全面贯彻党的十九大和十九届二中、三中全会精神，深入学习贯彻习近平总书记在辽宁考察时和在深入推进东北振兴座谈会上的重要讲话精神，统筹推进“五位一体”总体布局，协调推进“四个全面”战略布局，坚持稳中求进工作总基调，坚持新发展理念，坚持推动高质量发展，坚持以供给侧结构性改革为主线，深入推进突破辽西北发展战略，加快建设现代化经济体系，继续打好“三大攻坚战”，统筹推进稳增长、促改革、调结构、惠民生、防风险工作，坚定不移开展“重实干、强执行、抓落实”专项行动，聚焦补齐“四个短板”，全力推进“六项重点工作”，为全面建成小康社会收官打下决定性基础，加快推进铁岭全面振兴、全方位振兴，以优异成绩庆祝中华人民共和国成立70周年。

全市主要预期目标是：地区生产总值增长6.5%左右，一般公共预算收入增长7%，固定资产投资增长10%，规模以上工业增加值增长8%，社会消费品零售总额增长7%，城乡居民收入与经济增长基本同步。重点在以下6个方面实现新突破。

（一）以优化营商环境为基础，着力在重点领域和关键环节改革上实现新突破

坚持把深化改革作为铁岭振兴发展的关键一招，着力在谋划改革发展思路上下功夫，在解决突出矛盾问题上下功夫，在激发基层改革创新活力上下功夫，进一步坚定改革信心，做实改革举措，释放改革红利，提高改革效能。

全力打造营商环境最优市。牢固树立优化营商环境就是解放生产力、提高竞争力的理念。持续推进“一门、一网、一次、一窗”改革，工程建设项目审批时限压缩至90个工作日以内。深化“放管服”改革，深入推进“互联网+政务服务”，市县两级80%的政务服务事项实现“一网通办”。继续落实“项目管家”制度。加强中介服务事项清单管理。深入开展“兴企为民服务直通车”工作，再解决一批企业和群众“办事难”问题。加快推进“诚信铁岭”“法治铁岭”建设，有序化解历史遗留问题。在全市范围内开展“微笑服务”专项行动，进一步叫响铁岭质量、铁岭效率、铁岭服务、铁岭环境、铁岭品牌，以最优的营商环境，真正让广大投资者和老百姓安心、放心、舒心。

大力实施人才兴市战略。牢固树立人才是第一资源理念。落实推进人才集聚若干措施和人才服务全面振兴三年行动计划，加大高层次人才引进力度。加强现代职业技术教育，加大高素质劳动者和技术技能人才培养力度，努力培育一大批爱家乡、有本领的“铁岭工匠”。坚持关心本土人才与引进外来人才相结合，加快制定鼓励企业引才引智奖补政策，努力创造拴心留人的条件，让各类人才的智慧充分迸发，在铁岭振兴发展的舞台上大显身手！

全面深化国资国企改革。毫不动摇巩固和发展公有制经济，做强做优做大国有企业。按照“四个一批”的要求，加快推动国有企业战略性重组和混合所有制改革，建设科学规范的法人治理结构，加强内控体系和激励机制建设，深化企业三项制度改革，提升国有企业竞争能力。加强国有资产监管，建立覆盖全部国有企业的经营预算和收益分配制度。全面完成“三供一业”分离移交工作。按期完成厂办大集体改革任务。按照市场化、法制化要求处置僵尸企业，盘活存量资产。

大力发展民营经济。毫不动摇鼓励支持引导非公有制经济发展，进一步减轻企业税费负担，降低企业成本。帮助民营经济解决融资难、融资贵等实际困难和问题。加快诚信体系建设，构建亲清新型政商关系，打造公平竞争环境。积极落实支持民营企业发展的政策措施，鼓励民营企业参与国有企业改革，让民营企业从政策落实中增强获得感。深入实施“个转企、小升规、规升巨”培育行动，全年完成个转企工商户680户、小升规企业33户、规升巨企业9户。在全市开展“千人帮千企”专项行动，为民营企业提供差异化、定制化精准服务。积极鼓励大众创业、万众创新，为民营经济发展开辟绿色通道，让民营经济发展活力充分释放，让一切创造财富的源泉在铁岭大地充分涌流！

（二）以培育壮大新动能为抓手，着力在激发创新驱动内生动力上实现新突破

以建立现代化经济体系为目标，深入推进供给侧结构性改革，加快推进“一带四产业一区”建设，努力做好改造提升“老字号”、深度开发“原字号”、培育壮大“新字号”三篇大文章，培育发展新动能，加快形成新的产业支撑。

大力发展现代农业。继续深化农业供给侧结构性改革，坚持绿色发展，推进铁岭农业由大粮仓向绿色粮仓、绿色菜园、绿色厨房转变。坚持提质导向，调减籽粒玉米种植面积6.5万亩，新增设施蔬菜4200亩，提质改造高标准农田10万亩，主要农作物综合机械化水平达到89%，加快创建特色农产品优势区。深入实施农产品加工提升行动，推动农产品加工业向消费品工业转变，新建续建1000万元以上农业产业化项目10个。继续加大农业新型经营主体培育力度，加快农业生产标准化、规模化、国际化进程，重点推进榛子、苗木花卉、鹿茸等特色产业发展，打造一批信得过、叫得响的“铁”字号农产品品牌，全年新增省、市级名牌农产品12个以上。大力培育优势主导畜牧产业，持续抓好非洲猪瘟等疫病防控工作，新建改造规模化、标准化生态养殖场10个。完成国家级梅花鹿养殖标准化示范区建设，继续开展全国榛子产业和鹿产品知名品牌示范区创建工作。

深入实施工业强市战略。大力实施工业“提升、改造、盘活、上云、帮扶”五项工程，加快推进工业转型升级，促进制造业高质量发展。以传统能源产业向新型能源、绿色能源产业转变为方向，大力推进昌图30万吨燃料乙醇、神州集团保温材料等一批项目建设，加快建立安全低碳清洁高效的现代新能源体系。以特色装备制造业向先进装备制造业转变为方向，全力抓好丰源环保等30个高质量发展工业项目建设，实施长飞光缆三期等30个技术改造项目，引领装备制造业向中高端迈进。着力发展战略性新兴产业，重点培育发展智能制造、新材料、生物制药、数字经济、精细化工等新技术、新业态、新模式，构建产业发展新动能。新增高新技术企业10户，高新技术产品产值增长15%。大力推进军民融合产业发展，积极推动陆平东软移动CT诊疗车等项目建设，培育一批军工配套专业化承包商和供应商。深入实施名企、名牌、名家“三名”工程。开展闲置资产清查处置专项行动，全年嫁接改造停产企业、停建项目30个，盘活土地1000亩。

大力发展现代服务业。加快培育现代物流、文化、旅游、健康、养老产业，构建多元产业发展新支撑。以补齐发展短板和促进产业转型升级为导向，积极推进东北城二期项目建设，力争铁路物流基地投入使用，加快建设凡河新区现代物流核心区。强化金融广场功能，完善铁岭银行法人治理结构，继续抓好昌图农商行改制和银州清河农村信用联社整合改制工作。推动电子商务与一二三产业深度融合，打造优质电子商务平台。进一步整合旅游资源，加快推进蟠龙山旅游度假区、莲花湖湿地等项目建设。大力发展冰雪经济，抓好凡河新区雪域花灯“两湖一河”旅游项目建设，打造新城四季特色景观。全市旅游业总收入增长15%以上。

（三）以主动融入“一带一路”建设为引领，着力在推动全面对外开放上实现新突破

充分发挥辽宁“北大门”地缘优势，以战略眼光、全球视野谋划对外开放，积极融入“一带一路”建设，加快形成对外开放合作新格局。

持续开展项目年活动。坚定不移实施“双招双引”一号工程，聚焦产业基础和资源优势，围绕重点领域、重点区域、重点行业，深入开展抓招商上项目主题活动，全年到位内资120亿元以上，实际利用外资增速不低于全省平均水平。加快推进万达广场、碧桂园城市综合体、铁岭中电环保发电、清河区大数据产业园等重大项目建设。用好用足国家和省支持政策，储备亿元以上项目120个，争取各类政策资金135亿元以上。

主动融入全省对外开放平台。积极参与辽宁“一带一路”综合试验区和中国—中东欧“16+1”经贸合作示范区建设，深耕日韩俄，狠抓中东欧，充分利用俄罗斯、乌克兰等国家科技创新成果，举办辽宁（铁岭）独联体国家产业技术创新合作论坛，积极推进辽宁（铁岭）国际装备制造和新材料创新平台建设。依托铁岭保税物流中心，积极推进国际汽车产业贸易港、国际快件分拨监管中心、进出口商品集散中心建设，积极争取沈阳自贸区协作区政策支持，努力把铁岭建成中蒙俄经济走廊和丝绸之路经济大通道上的重要节点城市。

（四）以科学统筹精准施策为导向，着力在推进区域协调发展上实现新突破

把区域协调发展作为铁岭全面振兴、全方位振兴的工作重点，立足资源禀赋、功能定位差异，加快构建多极发力、多点支撑的区域发展新格局。

深入实施乡村振兴战略。认真落实乡村振兴战略三年行动计划。抓好农村土地“三权分置”改革，全面完成土地确权登记颁证工作。持续深化农村集体产权制度改革，加快农村综合产权交易市场建设。实施“清零递增行动”，力争有经济收入村达到75%以上。加大“四好农村路”建设力度。全力推进新三线三期建设。继续抓好农村人居环境整治、危房改造和“厕所革命”。积极促进农村一二三产业融合发展，规划建设一批田园综合体、旅游小镇、共享农庄等新产业新业态。

加快发展县域经济。把发展“飞地经济”作为破解乡镇项目落地瓶颈、壮大乡村集体经济的重要举措。进一步完善“飞地经济”相关政策措施，力争实现飞地项目所有乡镇全覆盖。完善特色乡镇建设规划，加快培育一批产业特色小镇。继续深化园区管理体制改革，加快推进西丰县、银州区、清河区工业园区晋级工作，努力实现省级开发区全覆盖。

建设美丽宜居城市环境。完成银州区玉深路、向阳泵站、二台子泵站建设和南排系统污水管网改造工程。积极推进银州区国道102线改建工程。大力实施城市基础设施维护养护、建筑工程质量安全提升工程，全面开展环境卫生、生活垃圾处理场和收集站、裸露土地绿化专项整治行动，提升城市精细化管理水平。有序推进县城、县域新城和中心镇建设，不断提升综合承载能力。进一步美化城市环境，提升城市品位，让人民群众过上安心、顺心、放心的好日子。

加强区域对接合作。主动与沈阳经济区、辽宁沿海经济带加强产业协作，深度参与京津冀协同发展。继续加强与淮安对口合作，促进干部人才交流、产业对接互补、合作平台共建，大力推进铁岭淮安工业园区建设，复制推广一批发达地区政策创新新举措。加快“铁四通辽”经济协作区建设，谋划好“西部大通道”项目，打造新的经济增长极。

（五）以全力打好“三大攻坚战”为重点，着力在夯实全面建成小康社会基础上实现新突破

继续打好防范化解重大风险、精准脱贫、污染防治“三大攻坚战”，针对突出问题，打好重点战役，确保如期完成任务目标。

有效防控各类重大风险。按照坚定、可控、有序、适度的原则，进一步加强政府债务管理，坚决遏制隐性债务增量，积极化解债务存量，确保政府债务风险可控，坚决守住财政支付风险底线。加大资金筹措力度，确保养老金按时足额发放。强化互联网金融、投融资等重点领域监管，依法严厉打击恶意逃贷行为，确保不发生区域性金融风险。

深入推进脱贫攻坚。聚焦持续、稳定脱贫，因地制宜发展壮大扶贫产业，实施产业扶贫项目234个。解决好因病致贫、因贫失学、贫困家庭学生就业等突出问题。完成大数据平台建设，进一步提升精准扶贫动态管理水平。充分发挥“收入保”“健康保”作用，确保18342名建档立卡贫困人口脱贫、42个贫困村退出，确保脱真贫、真脱贫。

大力推进污染防治。牢固树立绿水青山就是金山银山、冰天雪地也是金山银山的理念。深入实施生态建设“五大工程”，扎实开展重污染河流、非煤矿山企业等11个专项整治行动，全力抓好中央环保督察和“回头看”交办问题整改工作，严肃查处违法案件，强化追责问责。严格落实河长制，以超常规的举措推进辽河铁岭段流域治理。全力抓好“大棚房”专项整治，确保农地姓农。继续抓好秸秆综合利用，综合利用率达到86%以上。全市造林绿化11.3万亩，打造省级绿化示范村100个。继续完善土壤详查工作，进一步加强土壤污染防治。推广农村生活垃圾“五指分类法”，加快畜禽粪污资源化利用整县推进项目建设，切实改善农村人居环境。以前所未有的决心和力度加强生态环境保护，牢牢守住生态保护红线、环境质量底线、资源利用上线，让铁岭的天更蓝、水更清、山更绿、环境更美好！

（六）以补齐民生领域短板为方向，着力在幸福铁岭健康铁岭建设上实现新突破

牢固树立以人民为中心的发展思想，坚持尽力而为、量力而行的原则，着力解决好重点领域民生问题，让人民群众有更多、更直接、更实在的获得感、幸福感、安全感。

办好10件民生实事。1.全市城乡最低生活保障标准平均提高7%以上。2.新增城镇就业1.3万人。3.实施贫困残疾人家庭无障碍改造1150户。4.维修改造农村道路1250公里。5.建设美丽乡村示范村25个。6.改造银州区污水管网2125米。7.启动实施全市1175个行政村“雪亮工程”。8.改善20所农村中小学办学条件，30所农村普惠性幼儿园提质晋级。9.新建改造农村饮水安全工程124处，解决17.9万人饮水安全问题。10.城乡居民大病保险报销比例最高补偿标准由60%提高到75%。

提高收入水平和就业质量。深入实施城乡居民和各类群体收入增长三年行动计划。全面落实各项就业创业政策，城镇登记失业率控制在4.5%以内。不断完善社会救助体系，全面取消城乡低保县区差异，提高城乡居民医疗保险筹资标准，扩大保障范围。

大力发展各项社会事业。优先发展教育事业，努力办好人民满意的教育,让每个孩子都能享有公平而有质量的教育。建设连锁幼儿园20所，普惠性幼儿园覆盖率达到78%。继续推进铁岭师专现代服务业专业群项目建设。加强师德师风建设，进一步提高教师专业素养，规范校外培训机构发展。加快推进文化产业示范基地建设，繁荣文艺创作，推出一批文艺精品。加大文物和非物质文化遗产保护力度。举办好铁岭市第九届全民运动会。持续深化医药卫生体制改革。加快健康铁岭建设，推动市中心医院异地新建工程，积极创造条件，推进市综合性老年人养护中心尽快投入使用。高质量完成我市第四次全国经济普查工作。继续开展先进文化进万家和群众性精神文明创建活动，培育和践行社会主义核心价值观。

强化社会治理。深入开展扫黑除恶专项斗争，加快建立全民共建共治共享的社会治理体系。建立有效预防化解社会矛盾纠纷体制和就地解决群众合理诉求机制，加强校园及周边安全整治工作，严厉打击各类违法犯罪行为，推进平安铁岭建设。落实安全生产责任制，坚决遏制重特大安全生产事故。强化食品药品监管，深入开展农村假冒伪劣食品专项整治行动，确保群众吃得放心、用药安心。加强社区治理体系建设，实现政府治理和社会调节、居民自治良性互动，确保社会和谐稳定。

三、重实干、强执行、抓落实，建设人民满意政府

各位代表！新时代是奋斗者的时代。政府工作在新的一年里要有新气象新担当新作为，要牢固树立“四个意识”，坚定“四个自信”，坚决做到“两个维护”，加快推进政府职能转变，大力弘扬“严、细、深、实”的工作作风，为人民群众提供优质高效的服务。

打造实干政府。以“重实干、强执行、抓落实”专项行动为抓手，大力倡导务实精神，聚焦改革发展稳定中的突出问题，说实话、出实招、办实事、求实效，以最大的决心、最好的状态、最实的作风、最强的干劲，确保党中央国务院、省委省政府和市委各项决策部署落实落地。

打造诚信政府。坚持“新官既要理旧账，又不欠新账”，坚决做到信守承诺，政府作出的决定必须落实到位、作出的承诺必须如期兑现。继续梳理历史欠账，加大遗留问题解决力度。加强政务诚信建设，健全守信激励和失信惩戒机制，深入开展政府失信问题专项整治，严惩失信行为，以政府公信力取信于民。

打造法治政府。全面推进依宪施政、依法行政。严格遵守宪法和法律，把政府活动全面纳入法治轨道。依法接受市人大及其常委会法律监督和工作监督，自觉接受市政协民主监督，主动接受群众监督和舆论监督，让政府的权力在阳光下运行。

打造廉洁政府。严格落实全面从严治党主体责任，持续推进党风廉政建设和反腐败斗争。坚决贯彻落实中央八项规定精神，驰而不息整治“四风”，做到秉公用权、依法用权、廉洁用权、为民用权，实现干部清正、政府清廉、政治清明，努力打造风清气正、海晏河清的从政环境。

各位代表！实现铁岭振兴发展，是新时代赋予我们的重大使命，更是全市300万人民的殷切期盼。让我们更加紧密地团结在以习近平同志为核心的党中央周围，高举习近平新时代中国特色社会主义思想伟大旗帜，在中共铁岭市委的坚强领导下，撸起袖子加油干，以新气象新担当新作为，努力干出一片新天地，干出一个新铁岭，以优异成绩庆祝中华人民共和国成立70周年！
